# Mindfulness training for medical students in their clinical clerkships: two cross-sectional studies exploring interest and participation

**DOI:** 10.1186/s12909-015-0302-9

**Published:** 2015-02-25

**Authors:** Inge van Dijk, Peter LBJ Lucassen, Anne EM Speckens

**Affiliations:** 1 Department of Psychiatry, Radboud University Medical Center, Postbus 9101, 6500 HB Nijmegen, The Netherlands; 2 Department of Primary and Community Care, Radboud University Medical Center, Postbus 9101, 6500 HB Nijmegen, The Netherlands

**Keywords:** Medical students, Clinical clerkships, Mindfulness-based stress reduction, Interest, Participation, Psychological distress, Mindfulness skills, Online survey

## Abstract

**Background:**

So far, studies investigating Mindfulness Based Stress Reduction (MBSR) training in medical students are conducted in self-selected, pre-clinical samples, with modest response rates without collecting data on non-participants. This study first examines interest and participation rates of students starting their clinical clerkships. Second, it compares students interested in a mindfulness training with non-interested students and students participating in a trial on the effect of MBSR with non-participating students on levels of psychological distress, personality traits, cognitive styles and mindfulness skills.

**Methods:**

We examined two student samples from the Radboud University Medical Center, Nijmegen:

*Study 1* From March to December 2010 we performed a cross-sectional pilot-study among 4th year medical students starting their clinical clerkships, assessing interest in a MBSR training. We compared scores on the Brief Symptom Inventory, the Neo Five Factor Inventory and the Five Facet Mindfulness Questionnaire of interested students with those of non-interested students using t-tests with Bonferroni correction.

*Study 2* From February 2011 to August 2012 we invited 4th year medical students starting their clinical clerkships to participate in a randomized controlled trial (RCT) on the effectiveness of MBSR. We compared scores on the Brief Symptom Inventory, the Irrational Beliefs Inventory and the Five Facet Mindfulness Questionnaire of participating students with those of non-participants using t-tests with Bonferroni correction.

**Results:**

*Study 1*: Ninety-five out of 179 participating students (53%) were interested in a MBSR training. Interested students scored significantly higher on psychological distress (*p* = .004) and neuroticism (*p* < .001), than 84 non-interested students.

*Study 2:* Of 232 eligible students, 167 (72%) participated in our RCT. Participants scored significantly higher on psychological distress (*p* = .001), worrying (*p* = .002), problem avoidance (*p* = .005) and lower on mindfulness skills (*p* = .002) than 41 non-participants.

**Conclusions:**

Interest in mindfulness training and response rates in a RCT on the effectiveness of MBSR among clinical clerkship students are equal to (study 1) or higher (study 2) than in studies on pre-clinical students. Interested students and participants in a RCT reported more psychological distress and psychopathology related character traits. Participants scored lower on mindfulness skills.

## Background

As opposed to medical school being an inspiring environment to practice skills and develop a professional attitude, high workload and personal demands can change it into a stressful period. Medical students already report symptoms of psychological distress and burnout before graduating from medical school. They experience more psychological distress than age matched peers, with up to almost 50% reporting burnout related complaints [1,2].

The scientific literature reports an increase of psychological distress [3,4] and a decrease of self-reported empathy [5,6] and life satisfaction [7] during medical school. This is relevant for clinical practice, because stress during medical school seems to be predictive for a lower work satisfaction and more work related problems after graduation [8,9]. Physician distress contributes to a lower quality of patient care [10-12] and patient satisfaction [13-15].

As prevention is better than cure, there is a large potential benefit in teaching medical students how to learn resilience attitudes such as self-awareness of stress signals and unhelpful automatic responses [16].

An intervention that is currently upcoming for healthcare professionals to enhance their resilience attitudes and to reduce stress is Mindfulness Based Stress Reduction (MBSR) training. MBSR is an 8-week group training of 2.5 hours a week in which participants learn to focus their attention on the present moment by means of various exercises like meditation, psycho-education and practice integrated in daily activities. They are encouraged to change unhelpful automatic patterns, enhance self-care and adopt a non-judging attitude.

MBSR was originally developed to support patients suffering from chronic pain [17], but is currently offered to a broad public, existing of patients as well as healthy individuals. Meta-analyses of the effectiveness of MBSR in a variety of target groups, show medium effect sizes [18-20]. For professional practice in patient care, mindfulness training facilitates self-compassion, curiosity, self-reflection and a beginner’s mind that is open to new approaches [21-25]. Awareness of perfectionism and recognising a ‘helping and fixing mode’ are thought to contribute to the underlying process of change in healthcare workers [24].

Looking more specifically at cohort controlled and randomised controlled studies in medical students, six studies have been conducted so far [26-31]. Five with modest response rates varying from 18 to 40%, or unknown [28] and one study allocating a random group of students to the intervention or control condition *before* inviting them to participate in the trial, resulting in a response rate of 79% [31]. Three out of six strongly reduced the duration of the MBSR intervention [28-30] or used combined groups of medical students and nursing students [28] or psychology students [30]. The three remaining studies, a randomized controlled trial of Shapiro et. al. [26] and Erogul et. al. [31] and a prospective, cohort controlled study of Rosenzweig et. al. [27] used interventions closer to the classical MBSR training, targeting medical students only. All three showed positive results: students significantly improved in mood [27], psychological distress [26,31], self-compassion [31] and empathy [26]. In none of the above six studies, data were collected on non-participants and all studies concerned medical students in their pre-clinical phase.

In a recent paper summarising mindfulness based interventions in medical students, Dobkin and Hutchinson [32] conclude that although the evidence points to the usefulness of teaching mindful practices, various issues regarding timing, format and integration in the curriculum remain to be considered. In the present study, we aim to contribute to the existing knowledge by further exploring two questions raised by the above results from literature:

1. *Given the modest response percentages in pre-clinical medical students, how would interest in MBSR and participation in a RCT on the effectiveness of MBSR be among clinical clerkship students?*
  Clinical clerkships might be the time that the need for support is highest among students and the time that offers many opportunities to integrate exercises from the training in daily activities. Despite this, we expect interest in participation in a MBSR training to be lower among clinical clerkship students than among students in pre-clinical phase, because clinical clerkship students experience a higher workload and lower amount of leisure time. This might withhold them from applying for a training.
2. *Which medical students do we reach by offering a training?*

It is often suggested that, when making use of self-selected sampling, those who are more distressed will be more likely to apply for stress reducing interventions. Although this might seem logical, from current literature we do not have any information on characteristics of medical students interested in MBSR versus non-interested students or of medical students participating in a RCT versus non-participants. It could also be the case that students with higher amounts of psychological distress, feel unable to invest any extra time in a training or might be ashamed to participate. To answer this question, we will compare levels of psychological distress, personality traits and mindfulness skills in interested students to those of non-interested students and also in participants in a RCT investigating the effect of MBSR to those of non-participants.

## Methods

### Design, participants and procedure

We used two different student samples to answer our research questions. First, we performed a pilot study (study 1), a cross-sectional survey to examine interest in participation in a mindfulness training as part of exploring the feasibility of a RCT on the effect of MBSR in medical students in their clinical phase. We compared interested students to non-interested students on levels of psychological distress, personality traits and mindfulness skills. Second, we invited students to participate in the above mentioned RCT and collected baseline data on participants as well as non-participants (study 2).

### Study 1

We performed a cross-sectional survey from March to December 2010 among all 4th year medical students of the Radboud University Medical Center, Nijmegen at the start of their clinical clerkships. We offered students an interactive lecture on mindfulness-based interventions, which focused on current scientific literature on mindfulness-based interventions in psychiatry and included a guided mindfulness practice (bodyscan) and enquiry. The lecture was integrated in the core medical curriculum as part of the preparation period for their psychiatry internship, one of the first internships. It was presented by an experienced psychiatrist and mindfulness trainer. After the lecture students were asked if they were willing to complete a set of questionnaires on psychological distress, personality traits and mindfulness skills. As part of the questionnaire, they were asked if they would be interested in participating in a full eight-week MBSR training in case this would be offered to them in the near future. Students could receive feedback on their individual scores on the questionnaire, if they wanted to.

### Study 2

From February 2011 to August 2012 we invited 4^th^ year medical students at the start of their internships to participate in an RCT on the effectiveness of MBSR on psychological distress, aspects of well-being and aspects of professionalism. Information about the trial was provided after a lecture on physician wellbeing as part of the core medical curriculum. If students were interested in participation in the trial, they received an information letter by e-mail, giving them time to individually reconsider participation at home. Non-participants were asked if they would be willing to complete a onetime assessment, similar to the baseline assessment of trial participants. We collected information from participants and non-participants by means of an online survey, which students could access at home with a personalized link. Both participants and non-participants gave informed consent before completing the survey.

The MBSR training we offered was based on the classical training as developed by Kabat-Zinn et. al. [17] using an 8-week face-to-face program with formal exercises like a bodyscan, meditation and yoga next to informal practice to cultivate self-awareness.

### Measures

#### Study 1

The set of questionnaires that students completed in study 1 included the following measures:

- *Brief Symptom Inventory (BSI)*
  The BSI is a 53-item questionnaire, measuring psychological symptoms of distress in both clinical and non-clinical populations. It was developed as a short form of the 90 item Symptom Check List (SCL-90) [33]. A five point Likert scale is used to score items from ‘none-at-all’ to ‘extremely’. In our study, we used the global severity index (GSI). This is the mean score of all 53 items and is commonly used as a measure for overall psychological distress. The Dutch BSI has been found to have a high reliability and high validity [34,35].
- *Neo Five Factor Inventory (NEO-FFI)*
  The NEO-FFI measures five personality domains; neuroticism, extraversion, agreeableness, conscientiousness, and openness to experience. The NEO-FFI comprises 60 items, 12 for each domain. The internal consistency of the Dutch NEO-FFI was found to be acceptable to good on all domains (.64 to .88). The six month test-retest reliability and the construct validity are satisfactory [36].
- *Five Facet Mindfulness Questionnaire (FFMQ)*
  With the 39-item FFMQ five domains of mindfulness skills are assessed: observing, describing, acting with awareness, non-judging of inner experience and non-reactivity to inner experience [37]. Adding up the domains results in a total score of mindfulness skills. Items are rated on a 5-point Likert-type scale from ‘never or very rarely true’ to ‘very often or always true’. The subscales of the Dutch FFMQ have been shown to have good internal consistencies [38].

#### Study 2

In study 2, in addition to the FFMQ and BSI, we used the Irrational Beliefs Inventory (IBI) instead of the NEO-FFI. We expect the NEO-FFI to measure ‘trait’ personality characteristics, being rather stable over time. As the baseline measurement of study 2 is part of a longitudinal intervention study, we wanted to measure personality characteristics that we expect to be more reactive to change over time than those measured by the NEO-FFI. Therefore, we chose to use the Irrational Beliefs Inventory which measures cognitive styles instead.

- *Irrational Beliefs Inventory (IBI)*
  We used the five subscales of the 50-item IBI to assess students’ irrational cognitions, which are considered to be related to a person’s vulnerability for developing psychopathology [39]. The IBI is derived from the Irrational Beliefs Tests [40] and the Rational Behavior Inventory [41] but with improved psychometric quality. The subscales are worrying, rigidity, need for approval, problem avoidance and emotional irresponsibility. They are rated on a 5 point Likert scale from ‘strongly disagree’ to ‘strongly agree’. Reliability of the subscales and total scale was satisfactory (Cronbach’s alpha 0.70-0.85) [42].

### Statistical analysis

All data of study 1 were collected and anonymised in Microsoft Access and exported to IBM SPSS statistics 20.0. We collected data of study 2 using an online survey tool (Limesurvey) and exported them to IBM SPSS statistics 20.0 for anonymous analysis. The use of an online survey tool resulted in very few missing data. Missing data analyses revealed that they were missing completely at random (MCAR), therefore we did not use any imputation method. We used independent sample t-tests to compare mean scores between students interested in a mindfulness training and non-interested students (study 1) and between students participating in our trial and non-participants (study 2). We used Kolmogorov-Smirnov tests and visual inspection (Q-Q plots, histograms) to assess normality. If data were not meeting normality assumptions, we used non-parametric Mann–Whitney U tests. All statistical tests were performed two sided using an alpha of 0.05.We applied a Bonferroni-correction to correct for multiple testing and prevent excessive type I errors. Where possible, the magnitude of differences between compared groups was computed as standardized effect size measure (Cohen’s d). We considered values of 0.2 small, 0.5 medium and 0.8 large [43].

### Ethical considerations

In consultation with the ethical committee of the Radboud University Medical Center, study 1 was exempt from full ethical assessment because of limited effort asked from students. As part of a randomized controlled trial, data collection of Study 2 was fully assessed and approved of by the ethical committee of the Radboud University Nijmegen Medical Centre, protocol registration nr. 2010/388 and ABR nr.: NL33969.091.10.

## Results

### Study 1

#### Interest in a MBSR training and comparison of interested with non-interested students

**Figure 1:**
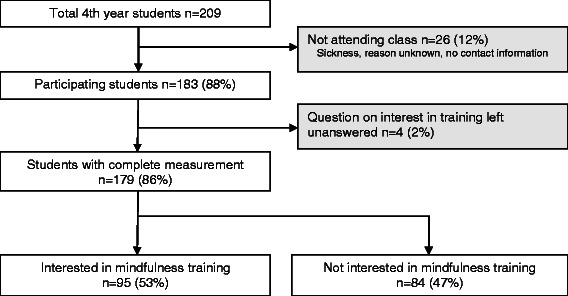
**Flowchart describing recruitment and participation of study 1.**

Of all 209 4^th^ year students, 179 (86%) completed the questionnaires of whom 95 (53%) were interested in following a MBSR training in case this would be offered to them in the near future (see Figure 1). There were no significant differences in mean age and gender between the 95 interested and 84 non interested students. However, interested students reported higher levels of psychological distress, neuroticism and agreeableness (see Table 1). After correcting for multiple testing (alpha level 0.05/9 = 0.0056) only the differences on psychological distress (*p* = .004) and neuroticism (*p* < .001) remained significant.

**Table 1:** **Study 1: Comparison of students interested and not interested in a mindfulness training**

	Total group (n = 179)	Interested in MFN (n = 95)	Not interested in MFN (n = 84)	Mean difference [95% CI]	P value ^ a ^	Cohens d
Age, median in years	22.0	22.0	22.0^b^		.17	
Female sex, n (%)	120 (66%)	66 (69.5%)	51 (61.4%)^c^		.26	
Psychological distress, median score:^d^	0.23	0.26	0.19		.004*	
Personality traits, mean scores (SD):						
Neuroticism *(range 12–60)*	28.9 (8.4)	30.9 (7.9)	26.6 (8.4)	4.2 [1.8;6.6]	<.001*	0.52
Extraversion *(range 12–60)*	43.9 (6.5)	43.5 (6.2)	44.3 (6.8)	−0.85 [−2.8;1.1]	.39	0.13
Openness to experience *(range 12–60)*	38.9 (5.1)	39.4 (5.3)	38.4 (4.8)	1.0 [−0.50;2.5]	.19	0.20
Agreeableness *(range 12–60)*	46.0 (4.7)	46.7 (4.8)	45.2 (5.5)	1.5 0.12;2.9]	.03	0.29
Conscientiousness *(range 12–60)*	44.0 (6.0)	43.5 (6.0)	44.5 (6.0)	−1.0 [−2.8;0.77]	.27	0.17
Mindfulness skills, total mean score (SD), *range 39–195*:	132.3 (13.0)	131.1 (13.4)	133.7 (12.5)^b^	−2.7 [−6.5;1.2]	.18	0.20

^a^alpha level after Bonferroni correction 0.05/9 = 0.0056.

^b^information not available for 2 students.

^c^information not available for 1 student.

^d^the median is reported because of skewed data and use of non-parametric Mann Whitney *U* test.

*statistically significant after correcting for multiple testing, p < 0.0056.

### Study 2

#### Participation in a trial on the effect of MBSR and comparison of baseline measurements of participants to non-participants

**Figure 2:**
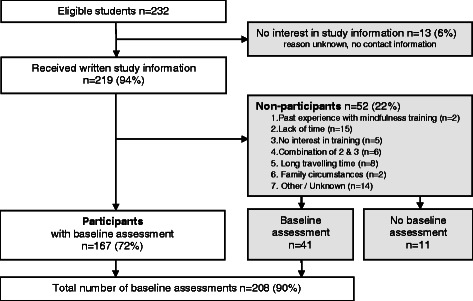
**Flowchart describing recruitment and participation of study 2.**

Of 232 eligible students, 167 (72%) students participated in the RCT, knowing that they could be randomized to receive the mindfulness training. Of 52 students who did not want to participate in the trial, 41 (79%) were willing to complete a onetime baseline assessment (see Figure 2), which resulted in a total 208 of baseline measurements (90%). Comparing participants with non-participants, we found no differences in age and gender. However, after applying a Bonferroni correction for multiple testing, participants still reported significantly more psychological distress (*p* = .001), worrying (*p* = .002) and problem avoidance (*p* = .005) and less total mindfulness skills (*p* = .002) than non-participants (see Table 2).

**Table 2:** **Study 2: Comparison of participants versus non-participants in the mindfulness trial**

	Total group (n = 208)	Participants (n = 167)	Non participants (n = 41)	Mean difference [95% CI]	P value ^ a ^	Cohens d
Age, median in years	23.0	23.0	23.2^b^		.92	
Female sex, n (%)	160 (76.9%)	131 (78.4%)	29 (70.7%)		.31	
Psychological distress, median score (SD)^#^	0.32	0.36	0.21^b^		.001*	
Cognitive styles, mean/median scores (SD)						
Worrying *(range 12–60)*^#^	34.0	35.0	29.0^c^		.002*	
Rigidity *(range 14–70)*	36.2 (5.6)	35.9 (5.6)	37.4 (5.4)^c^	−1.5 [−3.5;0.52]	.15	0.27
Need for approval *(range 7–35)*	23.5 (4.1)	23.7 (4.2)	22.8 (3.9)^c^	0.93 [−0.54;2.4]	.21	0.23
Emotional irresponsibility *(range 7–35)*	22.1 (3.7)	22.0 (3.8)	22.5 (3.3)^c^	−0.54[−1.9;0.78]	.42	0.15
Problem avoidance *(range 10–50)*	23.5 (5.2)	23.9 (5.3)	21.3 (4.1)^c^	2.7 [0.83;4.5]	.005*	0.56
Mindfulness skills, mean total score (SD), *range 39-195*	131.3 (14.5)	129.8 (14.6)	137.7 (12.5)^d^	−7.9 [−13.0;-2.9]	.002*	0.58

^a^alpha level after Bonferroni correction 0.05/9 = 0.0056.

^b^information not available for 1 student.

^c^information not available for 4 students.

^d^information not available for 3 students.

^#^The median is reported because of skewed data and use of non-parametric Mann Whitney *U* test.

*statistically significant after correcting for multiple testing, p < 0.0056.

## Discussion

In study 1, we found that 53% of students were interested in participating in a MBSR course. Interested students reported significantly higher levels of psychological distress and neuroticism, a measure of emotional instability, than non-interested students. As neuroticism is one of the key character traits in making people vulnerable to developing psychopathology such as anxiety, depression and burnout [36,44], it correlates with levels of psychological distress, therefore it is in line with current literature that neuroticism also differs between the groups.

In study 2, 72% of students participated in a randomized controlled trial of MBSR.

Participants of the trial reported significantly more psychological distress, worrying and problem avoidance than non-participants. Worrying and problem avoidance both correlate positively with psychopathology and neuroticism, thus making people vulnerable for psychological distress [42].

Furthermore, we found that participants reported less total mindfulness skills than non-participants which, assuming that students participating in a MBSR training will increase their level of mindfulness skills, seems to confirm that we reach those students who need it most, those with higher distress and lower skills.

### Interest in a mindfulness training

The 53% students interested in a mindfulness training at the start of their clinical clerkships (study 1) is higher than we expected based on the modest response percentages in pre-clinical students so far. Even if we would assume the worst case scenario that the 26 students who did not attend the lecture on mindfulness were all absent because they were not interested in the subject, the rate of interested students would still be 45%. The largest limitation of study 1 is, of course, that these students were only asked if they would be interested in following a training, so we do not know how many would have truly participated.

Looking at the results of study 2, the response percentage of participants in the trial (72%) is not only high compared to existing studies in pre-clinical students, but also to the percentage of interested students in study 1. Possibly, compared to pre-clinical students, experiences from their clerkships made students in their clinical phase more aware of the risks of psychological distress in their future residencies and of their own response to the high workloads. It at least does not seem the case that clerkships withheld them from participation.

A number of other factors could have contributed to the difference between the interest rate and participation rate in study 1 and 2:

First, part of the students might have participated because they think it is important to support research in general, not because they wanted to participate specifically in a MBSR. Second, students in study 1 and 2 were introduced to the study in different ways; the introduction in study 2 was more theoretical and students knew that they would have 50% chance to be randomized to receive the training. Third, it could be that students who would not have actively applied to follow a mindfulness training as elective course, did apply for participation in the trial because it was easily accessible or because they were just curious. And last, maybe part of the students favoured the 50% chance of following the regular curriculum instead of receiving the training, but took the risk of participation anyway. Still, taking all these factors in consideration, the response percentage remains high, which implies that even though clinical clerkship students are more busy, they also might be more in need of support than pre-clinical students. The high response rate also suggests that integration in the core curriculum could be feasible instead of offering an elective training.

### Interested vs. not interested and participants versus non-participants

Students interested in a training (study 1) and students participating in a trial (study 2) reported higher levels of psychological distress, neuroticism (study 1) and worrying (study 2) than non-interested and non-participating students. Both neuroticism and worrying make people more prone to developing psychopathology. This implies that we probably reach those who could potentially benefit most from the training.

Interestingly, the levels of psychological distress found in study 2 were higher than in study 1, even though the baseline assessments were conducted during the same period in medical curriculum at the start of clinical clerkships. Also, total mindfulness skills in study 2 differed significantly between participants and non-participants, which was not the case in study 1 between interested and non-interested students. These findings could possibly be explained by the fact that in study 1, students received an interactive lecture on mindfulness before completing the questionnaire, including a guided mindfulness practice (bodyscan) of 45 minutes. This could have lowered ‘state’ psychological distress and have influenced the score on mindfulness skills reported by the students. Another explanation to this difference could be found in the gender sensitivity of the instruments used. In general, women report higher levels of psychological distress and neuroticism than men. As the percentage of women in study 2 (77%) was higher than in study 1 (66%) this might have contributed to more pronounced differences between participants and non-participants in both studies. However, it should be noted that although our findings are statistically significant, the absolute differences are small and need further study to see if they are clinically relevant.

### Strengths and limitations

As far as we know, our study is the first to actively gather information on non-interested and non-participating students in the start of their clinical clerkships. Response rates were high in both study 1 (86%) and study 2 (baseline measurements of 90% of students), which contributes to the validity of our data. Furthermore, in study 2 we used an online survey program, which allowed students to complete the questionnaires at home in private, decreasing the risk of social desirable answers. A limitation of both studies is that they took place at only one medical centre in the Netherlands. We do not know whether results are generalizable to other medical schools. In addition, in study 1, students completed the questionnaire in a classroom, which could have led to social desirable answers possibly resulting in an underestimation of the level of psychological distress.

## Conclusions

In conclusion, interest and participation rates in clinical clerkship students were higher than found in current literature on pre-clinical students. This finding implies that the training could also be given during clerkships, possibly even resulting in better opportunities of integrating the exercises in hospital daily practice. Although our study seems to indicate that by offering the mindfulness training in regular medical curriculum we attract those students who could benefit most, those with higher levels of psychological distress and lower levels of mindfulness skills, it is still unclear what is the clinical relevance of this finding, as we did not use any outcome measures related to professional behaviour of students. This will have to be examined further in the future.
